# miR-21-mediated regulation of 15-hydroxyprostaglandin dehydrogenase in colon cancer

**DOI:** 10.1038/s41598-019-41862-2

**Published:** 2019-04-01

**Authors:** Nicholas J. Monteleone, Ashleigh E. Moore, Joseph R. Iacona, Carol S. Lutz, Dan A. Dixon

**Affiliations:** 1 Department of Microbiology, Biochemistry, & Molecular Genetics, Rutgers University – School of Graduate Studies, Newark, NJ 07103 USA; 2 University of Kansas Cancer Center, Kansas City, KS 66160 USA; 3 Department of Molecular Biosciences, University of Kansas, Lawrence, KS 66045 USA

## Abstract

Elevated prostaglandin E_2_ (PGE_2_) levels are observed in colorectal cancer (CRC) patients, and this increase is associated with poor prognosis. Increased synthesis of PGE_2_ in CRC has been shown to occur through COX-2-dependent mechanisms; however, loss of the PGE_2_-catabolizing enzyme, 15-hydroxyprostaglandin dehydrogenase (15-PGDH, *HPGD*), in colonic tumors contributes to increased prostaglandin levels and poor patient survival. While loss of 15-PGDH can occur through transcriptional mechanisms, we demonstrate that 15-PGDH can be additionally regulated by a miRNA-mediated mechanism. We show that 15-PGDH and miR-21 are inversely correlated in CRC patients, with increased miR-21 levels associating with low 15-PGDH expression. 15-PGDH can be directly regulated by miR-21 through distinct sites in its 3′ untranslated region (3′UTR), and miR-21 expression in CRC cells attenuates 15-PGDH and promotes increased PGE_2_ levels. Additionally, epithelial growth factor (EGF) signaling suppresses 15-PGDH expression while simultaneously enhancing miR-21 levels. miR-21 inhibition represses CRC cell proliferation, which is enhanced with EGF receptor (EGFR) inhibition. These findings present a novel regulatory mechanism of 15-PGDH by miR-21, and how dysregulated expression of miR-21 may contribute to loss of 15-PGDH expression and promote CRC progression via increased accumulation of PGE_2_.

## Introduction

Numerous studies have demonstrated the importance of prostaglandins in cancer progression, and that elevated prostaglandin E_2_ (PGE_2_) levels are associated with poor prognosis in various human malignancies, including colorectal cancer (CRC)^1–3^. Cyclooxygenases (COX) are the key enzymes involved in the synthesis of prostaglandins, and overexpression of the inducible isoform, cyclooxygenase-2 (COX-2, *PTGS2*) has been well established to occur during colorectal tumorigenesis^4–6^. Increased levels of COX-2-derived PGE_2_ have been shown to modulate several cancer-associated pathways including evasion of apoptosis, elevated tumor angiogenesis, cell proliferation, and migration^5,7,8^. The role of PGE_2_ in cancer progression has primarily focused on COX-2 dependent synthesis; however, PGE_2_ can be rapidly metabolized into its inactive form, and therefore its catabolism can also contribute to the amount of PGE_2_ present in tissues^9^. To this extent, a related pathway involving the degradation of PGE_2_ by 15-hydroxyprostaglandin dehydrogenase (15-PGDH; *HPGD*) has come to light as an essential mediator of prostaglandin levels^10^.

15-PGDH catalyzes the rate-limiting step of prostaglandin catabolism, and its inactivation has been shown to contribute to elevated levels of PGE_2_ in the colon^11,12^. 15-PGDH expression and activity is almost ubiquitously lost in human colorectal carcinomas as compared to matched normal tissue, and has also been demonstrated to be lost in colonic adenomas, indicating its importance during early neoplastic progression^12,13^. Additionally, reduced expression of 15-PGDH is associated with poor patient survival and correlates with a more aggressive cancer phenotype in gastric adenocarcinomas^14^. Genetic deletion of 15-PGDH in Apc^Min/+^ mice resulted in an approximate 8-fold increase in intestinal tumors, further indicating its tumor suppressive function in the gastrointestinal tract^12^. It has been reported that 15-PGDH expression is primarily lost through mechanisms involving epigenetic silencing and TGF-β signaling^13,15^. Interestingly, 15-PGDH mRNA also contains several regulatory sites present within its 3′ untranslated region (UTR), including AU-rich elements and putative microRNA-binding sites, implicating a role for post-transcriptional regulation in controlling 15-PGDH expression^2^.

MicroRNAs (miRNAs) are small non-coding RNAs, 18–22 nucleotides in length, that suppress gene expression post-transcriptionally^16^. miRNAs are fundamental regulators of gene expression and are predicted to regulate more than 60% of protein-coding genes^16^. miRNA expression has been shown to be dysregulated during tumorigenesis, thereby contributing to cancer progression due to mis-regulation of target mRNAs^17^. miR-21 is one of the most frequently upregulated miRNA in various human tumors, and is highly up-regulated miRNA in colorectal tumors^18–21^. Through this marked upregulation, circulating miR-21 has been shown to be an effective biomarker for early detection of CRC, along with a prognostic marker for aggressive disease, as it is associated with poor patient survival, advanced stage colorectal cancer, and reduced responsiveness to chemotherapy^21–23^. Through its pleiotropic upregulation in cancer and ability to regulate multiple tumor suppressor genes, miR-21 has gained much attention as a target for therapeutic inhibition^19,24–27^.

Resistance to chemotherapies poses a significant clinical hurdle, as most patients who initially respond to therapy eventually develop resistance^28^. One such mechanism is dysregulation of miRNAs^29^. Intriguingly, miR-21 has been shown to be upregulated in resistant cells, and inhibition of miR-21 resensitized cells to chemotherapeutics^26^. Additionally, miR-21 overexpression was associated with resistance to epidermal growth factor receptor (EGFR) inhibitors, including erlotinib^30^. It has also been shown that COX-2 and PGE_2_ contribute to resistance in CRC^31^. Targeted suppression of PGE_2_ production through NSAIDs and selective COX-2 inhibitors are effective in modulating prostaglandin levels and in preventing colon polyp recurrence; however, these treatment regimens are associated with thrombotic events and cardiovascular side effects^32^. Through its ability to regulate PGE_2_ levels, 15-PGDH is now being recognized as an alternative therapeutic target^33,34^, and elucidating the pathways that regulate its expression will be helpful in developing effective therapeutic strategies. The objective of this study was to determine if elevated miR-21 promotes constitutive repression of 15-PGDH expression, allowing for increased PGE_2_ levels observed in CRC tumors. These findings provide insights into the role of miR-21 as a novel post-transcriptional link between EGFR signaling and 15-PGDH expression that may lead to alternative therapeutic interventions to improve on CRC patient outcomes.

## Results

### miR-21 and 15-PGDH expression are inversely correlated

**Figure 1:**
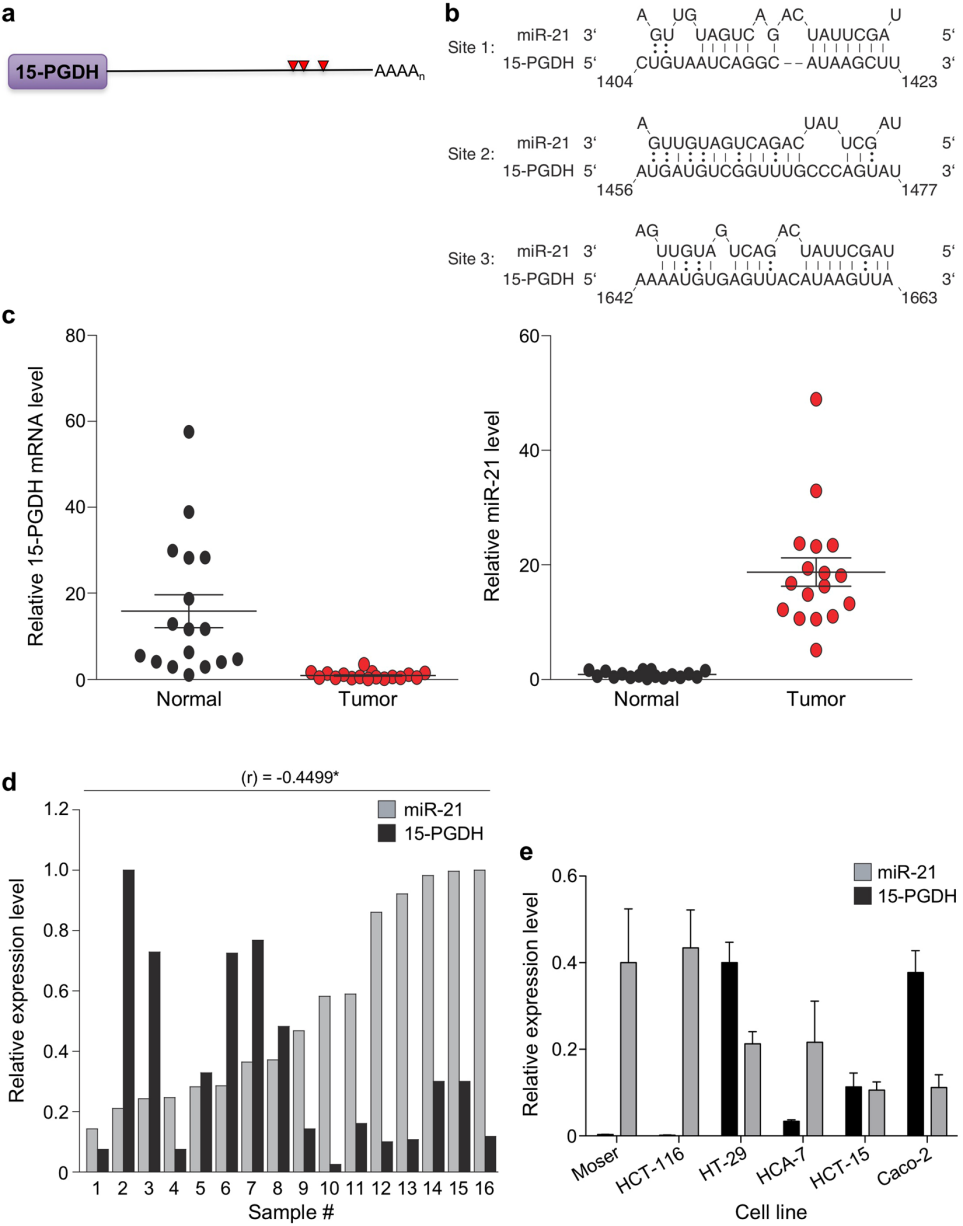
miR-21 and 15-PGDH expression are inversely correlated in CRC. (**a**) Illustration of the 15-PGDH 3′ UTR, including the position of miR-21 predicted binding sites (red). (**b**) Alignments of predicted miR-21 target sites in the 15-PGDH 3′UTR for each predicted sites by microRNA.org, RNAhybrid, and microCOSM, respectively. (**c**) Total RNA was isolated from matched normal colonic (grey) and tumor tissue (red, n = 16) and assayed for 15-PGDH mRNA and miR-21 expression levels. GAPDH and RNU6B served as internal controls, respectively. Fold change was determined by normalizing to the lowest 15-PGDH and miR-21 expression samples. (**d**) Correlation studies for normal colon tissue samples were normalized to the highest miR-21 (grey bars) or 15-PGDH (black bars) expressing sample. Correlation graph was plotted from lowest miR-21 expressing sample to highest, along with its respective 15-PGDH expression (*r = −0.4499, *P = 0.0038). (**e**) qPCR analysis of relative miR-21 (gray) and 15-PGDH (black) mRNA levels in 6 colorectal cancer cell lines (Moser, HCT-116, HT-29, HCA-7, HCT-15, Caco2) (r = −0.62, *P* = 0.19, n = 3).

miR-21 has been shown to be a consistently upregulated miRNA in all solid tumors, including CRC^21^. 15-PGDH serves as a tumor suppressor in gastrointestinal cancers, and is underexpressed in the majority of colon adenocarcinomas with low expression correlating to an aggressive disease phenotype^13^. Our prior work had identified that the 15-PGDH 3′UTR harbors putative miR-21 binding sites^2^, suggesting that elevated miR-21 in CRC may serve to inhibit 15-PGDH expression. Illustrated in Fig. 1a,b, three potential miR-21 binding sites were predicted within the 15-PGDH 3′UTR by *in silico* target prediction using RNAhybrid, microRNA.org, and microCOSM^35–38^.

**Figure 2:**
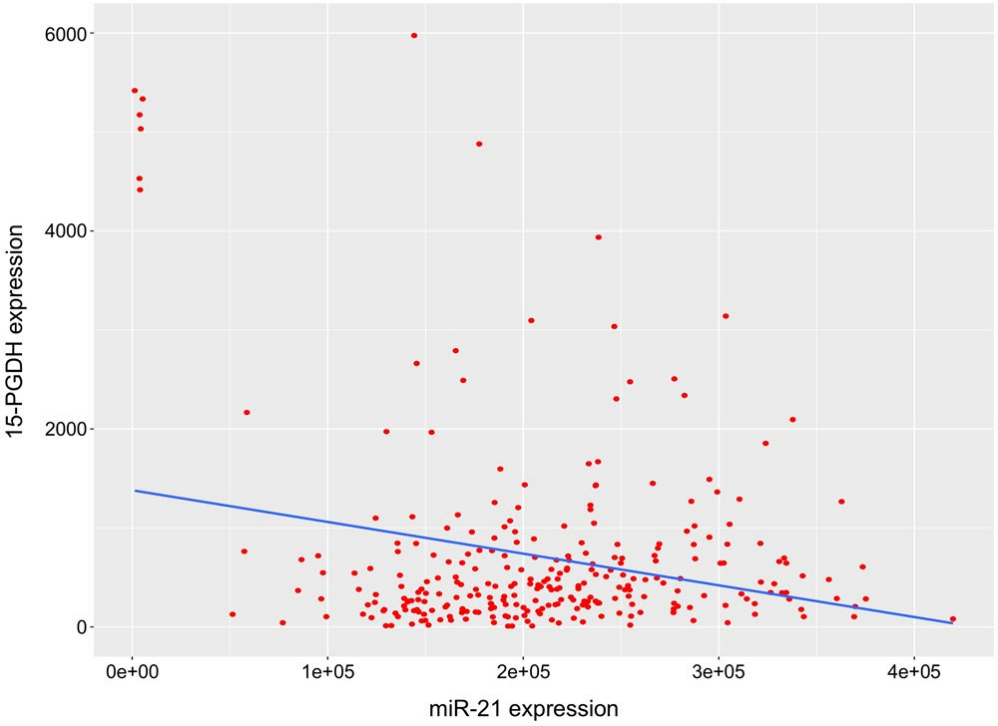
TCGA analysis of miR-21 and 15-PGDH expression in CRC. Scatter plot showing miR-21 levels (x-axis) vs. paired 15-PGDH mRNA expression (y-axis) from TCGA data. Red dots represent individual patients, while the blue line is a linear regression analysis (r = −0.23, *P < 0.00001, n = 287).

In order to determine if an inverse correlation between miR-21 and 15-PGDH expression existed *in vivo*, normal human colonic tissue and colon tumor samples were analyzed for 15-PGDH and miR-21 expression levels (Fig. 1c). 15-PGDH mRNA levels were attenuated in 94% of tumor samples with an average 17-fold decrease in 15-PGDH mRNA expression in tumor samples as compared to their matched normal colonic tissue (left panel). Conversely, miR-21 levels were shown to be elevated in 100% of tumor samples with an average 20-fold increase in miR-21 expression in tumor samples as compared to its matched normal colonic tissue (right panel). In normal colonic tissue, a significant inverse correlation of miR-21 levels with 15-PGDH mRNA expression was observed (r = −0.4499, *P = 0.0405) (Fig. 1d). 15-PGDH and miR-21 expression levels in tumor samples showed a similar trend of being inversely correlated, but was not found to be statistically significant (data not shown). To determine if this inverse correlation was also observed in colon cancer cell lines, a panel of colon cancer cell lines were analyzed for 15-PGDH mRNA expression and miR-21 levels. CRC cell lines (Moser, HCT-116, HT-29, HCA-7, HCT-15, Caco2) revealed a similar trend, although miR-21 levels and 15-PGDH mRNA expression were not significantly negatively correlated (Fig. 1e, r = −0.62, *P* = 0.19). Additionally, RNA-Seq and miR-Seq data from The Cancer Genome Atlas (TCGA) revealed miR-21 expression was significantly negatively correlated with 15-PGDH mRNA expression in colorectal cancer (r = −0.23, *P* < 0.00001, n = 287) (Fig. 2). Based on this data, we next examined if miR-21 could regulate 15-PGDH expression in CRC.

### miR-21 regulates 15-PGDH expression by directly targeting the 15-PGDH 3′UTR

**Figure 3:**
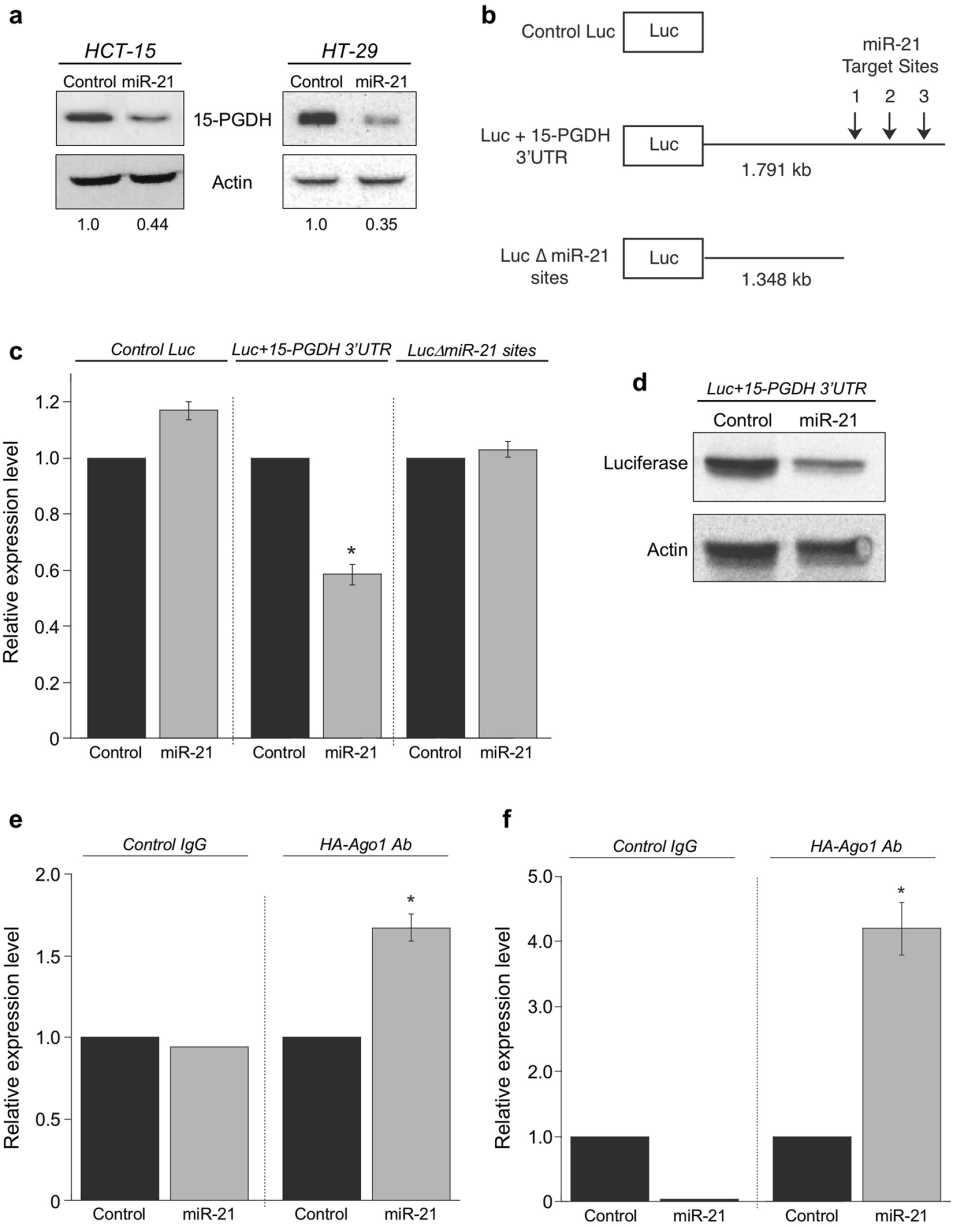
miR-21 suppresses 15-PGDH by directing targeting the 15-PGDH 3′UTR. (**a**) HCT-15 and HT-29 cells were transfected with miR-21 or control miR for 48 hours, after which 15-PGDH protein expression was assayed by western blot. Actin served as a loading control. Relative quantification of 15-PGDH normalized to actin is located below each western blot. (**b**) Luciferase reporter constructs without the 15-PGDH 3′UTR (Control Luc), or fused to the full-length 15-PGDH 3′UTR (Luc + 15-PGDH 3′UTR), or the miR-21 target sites deleted from the full-length 3′UTR (Luc Δ miR-21 sites). (**c**) HeLa cells were co-transfected with 3′UTR reporter constructs and miR-21 (grey bars) or control miR (black bars) for 48 hours. Luciferase activity was normalized to total protein and is the average of 3 experiments, (*P = 0.0014). (**d**) Western blot of luciferase and 15-PGDH protein expression from lysates of cells co-transfected with Luc + 15-PGDH 3′UTR and miR-21 or control miR; Actin served as a loading control. (**e**,**f**) HCT-15 cells were co-transfected with HA-tagged Ago1 expression plasmid and miR-21 (grey bars) or control miR (black bars) for 24 hours. (**e**) mRNP-IP of HA-Ago1 or control IgG was performed following analysis of 15-PGDH mRNA (*P = 0.0127). (**f**) mRNP-IP of HA-Ago1 or control IgG was performed following analysis of PTEN mRNA (*P = 0.0015).

In order to determine if miR-21 directly regulates endogenous 15-PGDH expression in colon cancer, HCT-15 and HT-29 colon cancer cells were transfected with synthetic miR-21 or control miR for 48 hr followed by 15-PGDH protein analysis. We chose HCT-15 and HT-29 cells because they express relatively low endogenous miR-21 (Fig. 1e), and both cell lines endogenously express 15-PGDH at the mRNA (Fig. 1e) and protein level (Fig. 3a). As shown in Fig. 3a, miR-21-transfected cells displayed decreased 15-PGDH protein expression as compared to control-transfected cells. To validate miR-21 targeting of the 15-PGDH 3′UTR, luciferase reporter constructs bearing the full-length 15-PGDH 3′UTR (Luc + 15-PGDH 3′UTR) and the miR-21 target sites deleted (Luc Δ miR-21 sites) were generated (Fig. 3b) and transiently co-transfected in HeLa cells along with miR-21 or control miR for 48 hr. Shown in Fig. 3c, miR-21 overexpression resulted in a 1.7-fold attenuation of luciferase activity in cells containing reporter constructs bearing the full-length 15-PGDH 3-UTR. This effect of miR-21 was not observed with the predicted miR-21 target sites deleted from the 15-PGDH 3′UTR, indicating this region as essential for miR-21-mediated regulation of 15-PGDH (Fig. 3c). Consistent with these results, miR-21 inhibited luciferase protein levels > 2-fold in cells containing the Luc + 15-PGDH 3′UTR reporter, along with inhibiting endogenous 15-PGDH protein levels (Fig. 3d).

To determine if miR-21 directly binds endogenous 15-PGDH transcripts, miRNA ribonucleoprotein immunoprecipitation (mRNP-IP) were performed. HCT-15 cells were cotransfected with miR-21 or control miR, along with an HA-tagged Argonaute-1 (HA-Ago1) expression vector to immunoprecipitate the miRISC that directs miRNA-mRNA interactions^16^. The association of 15-PGDH mRNA with HA-Ago1 was then assayed by qPCR of 15-PGDH mRNA in immunoprecipitates. As shown in Fig. 3e, 15-PGDH mRNA was significantly enriched in the HA-Ago1 immunoprecipated samples where miR-21 was over-expressed. As a control, an established target of miR-21, PTEN^39^, was also significantly enriched in miR-21/HA-Ago1 immunoprecipitates (Fig. 3f). Taken together, these results indicate that miR-21 can directly regulate 15-PGDH expression through its 3′UTR by association with miRISC.

### miR-21 regulates prostaglandin accumulation

**Figure 4:**
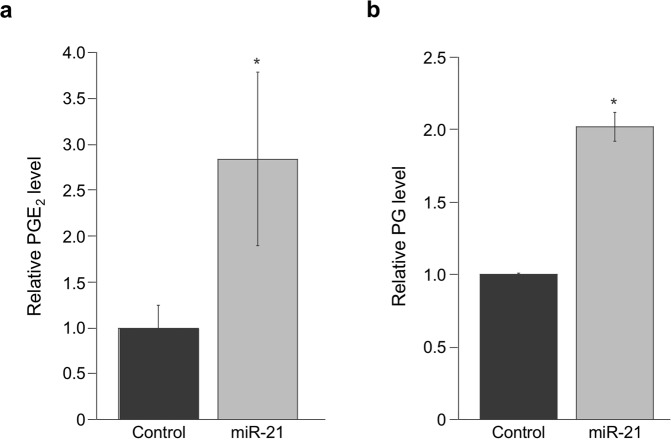
miR-21 increases PGE_2_ levels. 15-PGDH catabolic activity was evaluated by (**a**) PGE_2_ levels and (**b**) total PG production in HCT-15 cells transfected with miR-21 (grey bars) or control miR (black bars). Relative PGE_2_ and PG levels were normalized to total protein levels and are an average of 2 experiments (*P = 0.0046, *P = 0.0101).

Increased levels of PGE_2_ observed in CRC tumors have been shown to influence several cancer-associated pathways. Based on 15-PGDH’s role in catabolizing PGE_2_ and other prostaglandins (PGs)^9^, and that miR-21 can attenuate 15-PGDH protein expression, we hypothesized that miR-21 could promote elevated PG levels. To test this, HCT-15 cells that show low endogenous miR-21 levels and miR-21-mediated suppression of 15-PGDH expression (Figs 1e and 3), were transfected with synthetic miR-21 or control miR followed by assessment of prostaglandin levels in culture media. In miR-21-transfected HCT-15 cells, an approximate 3-fold and 2-fold increase in PGE_2_ and PG levels were observed, respectively (Fig. 4a,b), indicating that miR-21 can modulate prostaglandin production through regulating 15-PGDH protein expression.

### EGFR signaling regulates miR-21 and 15-PGDH

**Figure 5:**
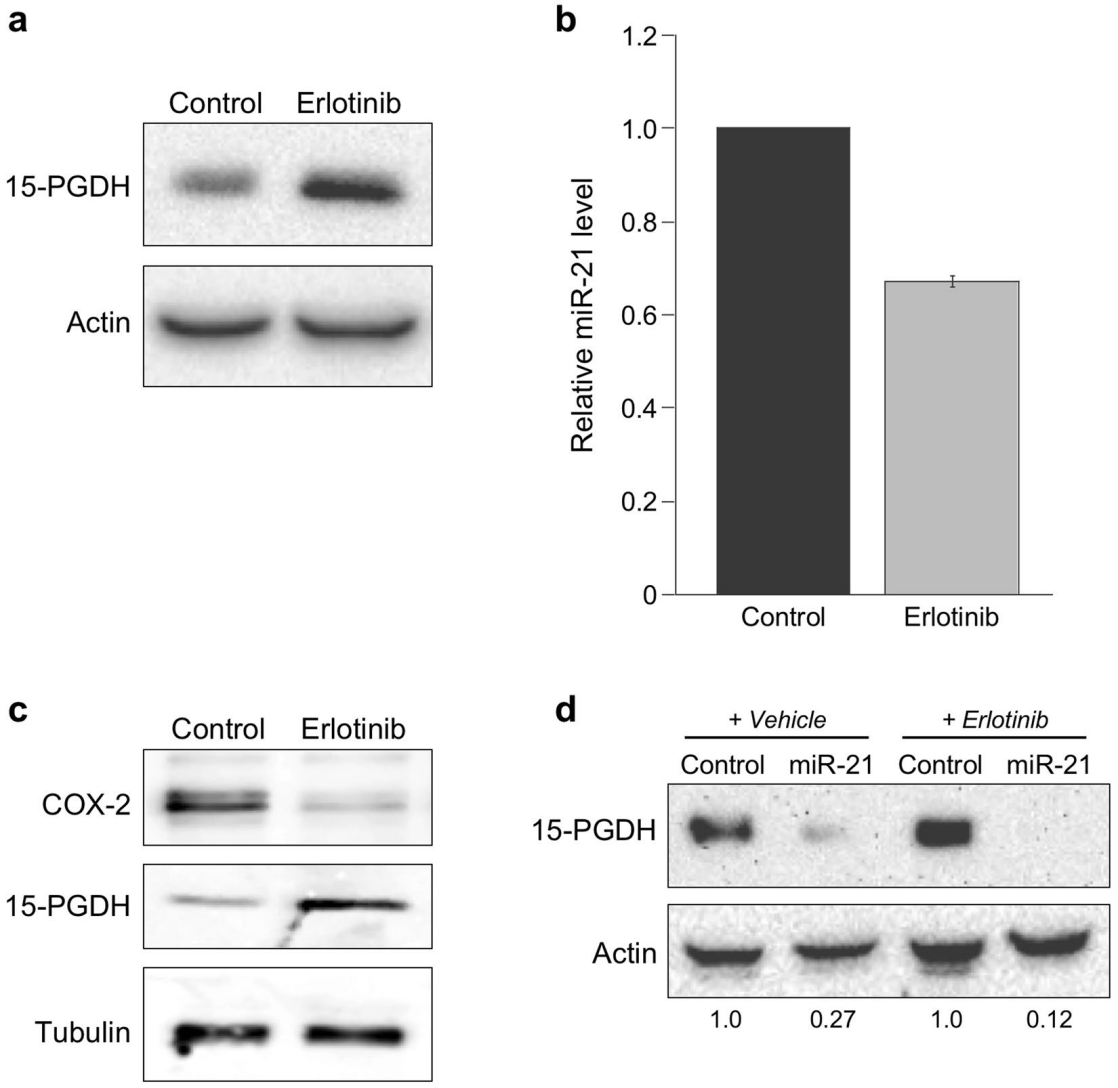
EGFR signaling regulates miR-21 and 15-PGDF. HT-29 cells were serum starved for 16 hr, following exposure to 100 ng/mL EGF for 1 hr, after which cells were treated with 5 μM Erlotinib or vehicle control for 5 hours and analyzed for (**a**) 15-PGDH protein, (**b**) relative miR-21 levels by qPCR, and (**c**) 15-PGDH and COX-2 protein expressions. Actin and tubulin served as protein loading controls; RNU6B served as an RNA internal control. (**d**) HT-29 cells were transfected with miR-21 or control miR for 48 hours, following serum starvation for 16 hr. Cells were then exposed to 100 ng/mL EGF for 1 hr, after which cells were treated with 5 μM Erlotinib or vehicle control for 5 hours and analyzed for 15-PGDH protein expression. Actin served as a loading control.

15-PGDH has been shown to be reactivated in colon cancer cell lines by inhibiting EGFR signaling^13,15,40^. Interestingly, other studies have shown that miR-21 expression is attenuated in response to EGFR inhibition^41^, indicating a potential miR-21-mediated mechanism for the observed increased 15-PGDH expression in response to EGFR tyrosine kinase inhibitors. To evaluate this, HT-29 cells were treated with the EGFR inhibitor erlotinib or vehicle control, and assayed for 15-PGDH protein levels and miR-21 expression. In cells treated with erlotinib, increased 15-PGDH protein levels as well as decreased miR-21 expression were observed (Fig. 5a,b). Additionally, erlotinib decreased COX-2 protein levels (Fig. 5c). To determine if erlotinib-dependent induction of 15-PGDH is due to its effect on decreased miR-21 levels, HT-29 cells were transfected with miR-21 prior to treatment with erlotinib or vehicle control. As shown in Fig. 5d, erlotinib-dependent induction of 15-PGDH was attenuated in the presence of miR-21, further implicating miR-21 as a post-transcriptional link influencing 15-PGDH levels downstream of EGFR signaling.

### miR-21 inhibition regulates 15-PGDH levels and cell proliferation rates

**Figure 6:**
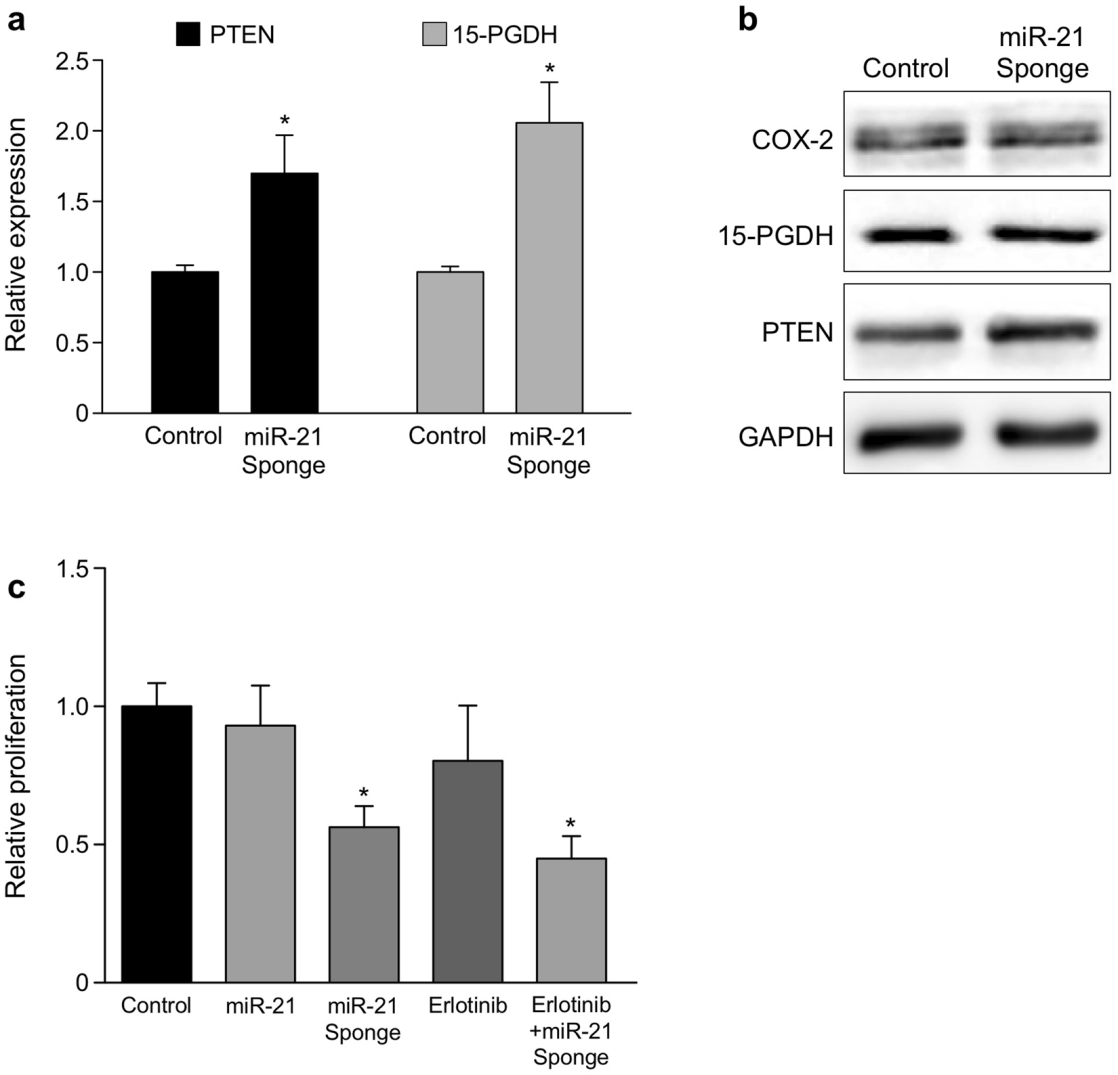
miR-21 inhibition regulates 15-PGDH levels and cell proliferation. (**a**) qPCR analysis of 15-PGDH and PTEN mRNAs after 48 hour transfections of the miR-21 sponge in HCT-116 cells. Data was normalized to U6 RNA (*P < 0.05, n = 3). (**b**) Western blot analysis of HT-29 cells transfected with the miR-21 sponge for 48 hr and probed for COX-2, 15-PGDH, and PTEN; GAPDH as a loading control. **(c)** WST-1 cell proliferation assay of HT-29 cells after 48 hr treatments of control miR, miR-21, miR-21 sponge, erlotinib, or erlotinib + miR-21 sponge. Data was normalized to total protein concentration (*P < 0.01, n = 3).

To establish if inhibition of miR-21 can reciprocally restore 15-PGDH expression, a miR-21 sponge was used in HT-29 and HCT-116 cells, as they express intermediate and high levels of miR-21, respectively. miRNA sponges act as decoy 3′ UTRs to sequester specific miRNAs, preventing the miRNA from binding to its endogenous targets^42^. The miR-21 sponge construct contains the GFP open reading frame under the control of the CMV promoter, while the 3′ UTR has multiple tandem miR-21 binding sites inserted^43^. These miR-21 binding sites have a three base mismatch within the middle portion of the sequence, producing a bulge that protects against endonucleolytic cleavage. In miR-21 sponge-transfected HCT-116 cells, elevated expression of both miR-21 target mRNAs PTEN and 15-PDGH were observed (Fig. 6a). Additionally, the miR-21 sponge increased 15-PGDH and PTEN protein levels (30% and 40%, respectively) in HT-29 cells, but did not change COX-2 expression (Fig. 6b). Lastly, we measured differences in HT-29 cellular proliferation after inhibition of miR-21 and EGFR signaling. Expression of the miR-21 sponge significantly reduced proliferation in HT-29 cells, whereas transfection of miR-21 or erlotinib alone did not impact proliferation significantly (Fig. 6c). Combination of erlotinib and the miR-21 sponge showed an additive effect in suppressing proliferation rates, suggesting potential synergy of EGFR and miR-21 inhibition in modulating CRC cell growth.

## Discussion

The relevance of PGE_2_ in colorectal cancer is well established, as elevated levels of PGE_2_ from colon cancer patients correlates with poor prognosis^7,8^. While overexpression of COX-2 is considered a primary means for increased PG synthesis in CRC, loss of 15-PGDH expression in colonic tumors is now recognized as a contributing factor to increased PGE_2_ levels. These changes in expression can result from various regulatory mechanisms, including the differential expression of miRNAs observed in colorectal tumors. COX-2 has been shown to be regulated through miRNA-mediated mechanisms^2,44–47^, however the potential of 15-PGDH to be regulated by miRNAs in CRC is not known. The findings presented here provide an additional level of understanding how control of PGE_2_ levels can occur through miR-21-mediated regulation of 15-PGDH.

Expression patterns of miRNAs are altered in colon adenocarcinomas, and miR-21 is the highest expressed miRNA in a wide range of solid tumor including CRC^18–21,48^. A direct correlation between elevated tumor miR‐21 expression and CRC is associated with worse clinical outcome^49^. Using clinical CRC samples and TCGA data sets, we show that miR-21 and 15-PGDH mRNA expressions were significantly negatively correlated in paired tumors, and in normal colonic tissue the inverse of this was observed (Fig. 1). We also analyzed miR-21 and 15-PGDH expression levels in colon cancer cell lines and saw a similar trend with our clinical data.

Colorectal tumors arise as a result of the activation of oncogenes as well as inactivation of tumor suppressor genes^50^. miR-21 is considered an oncomiR due to its constitutive overexpression in solid malignancies presumably through its ability to downregulate several tumor suppressors, metastatic, and apoptotic genes that have been identified as miR‐21 targets^51^. Our work extends on this list to include 15-PDGH as a miR-21 target in CRC, and are consistent with current observations in other tumor types where miR-21 was identified to be a regulator of 15-PGDH^52–55^.

Inhibition of COX-2 activity has been associated with adverse cardiovascular side effects, highlighting the importance of developing alternative therapeutic strategies to regulate prostaglandin levels^32^. 15-PGDH expression is lost in the majority of colorectal adenocarcinomas, and this loss is associated with altered prostaglandin levels and poor prognosis^12–14^, indicating that re-establishment of 15-PGDH expression may provide a therapeutic benefit. We show that inhibition of miR-21 using a deliverable miRNA sponge increased 15-PGDH levels and significantly reduced CRC cell proliferation rates (Fig. 6). This decrease in proliferation is consistent with observations using COX-2 specific inhibitors^56,57^ and provides an alternative therapeutic strategy to specifically target PGE_2_ levels in CRC tumors. Clinically, miR-21 has been implicated in resistance to chemotherapies, including EGFR inhibitors^26,30^. COX-2 and PGE_2_ also have a role in CRC resistance, and prostaglandin inhibition synergizes with EGFR inhibitors^31,32,58^. Our work supports these findings by showing a miR-21 sponge used in combination with erlotinib decreased CRC cellular proliferation greater than either therapy alone (Fig. 6). It would be worthwhile to investigate whether miR-21 inhibition can overcome erlotinib resistance, as this is a major obstacle with EGFR inhibitors in the clinic. Furthermore, selective inhibition of miR-21 may offer a greater effect on suppressing tumor growth, based on its ability to downregulate various tumor suppressor genes and promote resistance mechanisms. Therefore, it is appealing to determine the ability of miR-21 inhibition to synergize with conventional chemotherapies both *in vitro* and *in vivo*. This may be a more potent intervention than combination therapies using COX-2 specific inhibitors, as miR-21’s oncogenic qualities are not limited to arachidonic acid signaling.

There are several hurdles to climb before miRNA inhibitors can be used in CRC patients. The major obstacle is delivery, as it is crucial to get miRNA inhibitors into the tumor to ensure limited off-target effects at therapeutic concentrations^59^. Recently, clinical trials have provided hope for RNA therapeutic potential in cancer, and novel tumor targeting approaches are enhancing delivery of RNA molecules^60,61^. It is also critical to understand what effects miR-21 inhibition will have within the tumor microenvironment. While COX-2 and PGE_2_’s roles in tumor microenvironment are fairly well-defined^62^, much less is known about miR-21 and 15-PGDH. Current research has begun to develop therapeutics that modulate the tumor microenvironment composition, with some therapies including miRNAs^63,64^. Thus, it will be important to study how miR-21 functions within the tumor microenvironment, and how modulation of miR-21 could potentially reprogram the tumor microenvironment to influence tumor growth.

**Figure 7:**
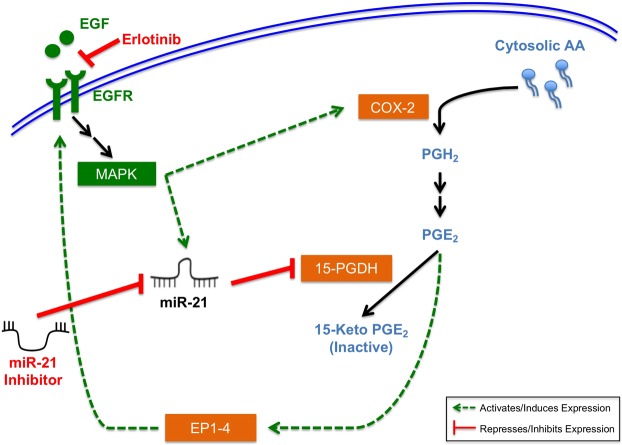
Proposed mechanism for coordinated regulation of miR-21, EGF, and prostaglandin signaling in CRC. Green dotted lines represent activation signaling cascades, red solid lines represent repressing mechanisms, and black lines indicate metabolic reactions. PGE_2_ can act in an autocrine or paracrine manner to activate PGE_2_ receptors EP1-4, which can then transactivate EGFR. Acroynyms: MAPK (Mitogen-Activated Protein Kinase), EP1-4 (Prostaglandin E2 Receptors 1–4), AA (Arachidonic Acid), PGH_2_ (Prostaglandin H2).

Loss of 15-PGDH expression and increased levels of miR-21 have both been reported to occur at the adenoma stage^12,13,65–67^, suggesting these changes may be an early cellular event during colorectal tumorigenesis occurring concomitantly. Previous work has shown that loss of 15-PGDH expression in CRC can be through transcriptional mechanisms, and 15-PGDH expression can be reactivated by correcting TGF-β or EGFR signaling^13,15,40^. Here we show that miR-21 expression is attenuated in response to EGFR signaling inhibition, indicating a potential mechanism for the observed increased 15-PGDH expression in response to EGFR inhibition^41^. PGE_2_ has also been shown to transactivate EGFR allowing for increased prostaglandin signaling and tumor cell growth^68^, which could potentiate the effect of dysregulated 15-PGDH and miR-21 expression. We propose a model by which miR-21, the EGF pathway, and arachidonic acid signaling interact to promote CRC tumorigenesis (Fig. 7). We acknowledge our model’s simplification, given the complex cross-talk between EGFR and arachidonic acid signaling, along with the pleiotropic effects of miR-21. Further work in completely defining this mechanism, as well as other miR-21-governed regulatory networks will further our understanding of this molecular pathway. Taken together, our findings indicate that miR-21 sits at the center of a molecular network and contributes to colon cancer progression in part through its ability to modulate PGE_2_ levels through regulating 15-PGDH expression.

## Methods

### Cell culture, DNA transfection, miRNA, and miRNA sponge transfection

HeLa, HCT-15, LS174T, HT-29, Caco2, and HCT-116 were purchased from American Type Culture Collection (ATCC, Manassas, VA). Moser cells were kindly provided by R.D. Beauchamp (Vanderbilt University Medical Center, Nashville, TN). HeLa, LS174T, HT-29, and HCT-116 cells were maintained in Dulbecco’s modified Eagle’s medium (DMEM) containing 10% FBS (Hyclone/ThermoFisher), 2mM L-glutamine, and 1% Pen-Strep (Gibco/Thermo Fisher). HCT-15 cells were maintained in RPMI medium containing 10% FBS, 2mM L-glutamine, and non-essential amino acids (Gibco/Thermo Fisher) and culture on 3% gelatin-coated plates. Caco2 cells were maintained in RPMI medium containing 20% FBS, 2mN L-glutamine, and 1% Pen-Strep. Luciferase reporter constructs containing the 15-PGDH 3′UTR was created by cloning the 15-PGDH 3′ UTR into the pcDNA3.1/Zeo(+) vector (Invitrogen, Carlsbad, CA) containing the luciferase cDNA as previously described^69^. The 15-PGDH 3′UTR was amplified from HeLa cDNA using the following Apa1-tagged primers, sense 5′-GGGCCCACAGCTTATGTGTTAGCCATAGCTG-3′ and antisense 5′-GGGCCCCCCCTCCCCTAACTTCAGTTTA-3′. Luc + 15-PGDH 3′UTR was digested with Xba1 to exclude the miR-21 target sites and ligated back into pcDNA3.1/Zeo containing the luciferase cDNA to create the LucΔmiR-21 sites construct. Transient transfections of cells with luciferase reporter constructs or HA-tagged Ago1 expression plasmid (pHA-Ago1)^70^ were accomplished using Lipofectamine Plus (Invitrogen) according to the manufacturer’s protocol. After 3 hours, the media was changed and cells were sequentially transfected with miRNA for 48 hours. MicroRNA transfection of cells using 50 nM hsa-miRNA-21 mature miRNA duplex or random sequence negative control miRNA #2 (Ambion, Austin, TX) were performed using siQuest (Mirus, Madison, WI) for 48 hours according to the manufacturer’s instructions. The miR-21 sponge construct (CMV-d2eGFP-21, Addgene, Watertown, MA)^43^ was transfected into HCT-116 cells using LipoD293 (SignaGen, Rockville, MD) for 48 hours, followed by RNA isolation using Trizol reagent.

### RNA analysis

Total RNA was extracted using Trizol reagent (Invitrogen). cDNA synthesis was performed using 1 µg of total RNA in combination with oligo(dT) and Improm-II reverse transcriptase (Promega, Madison, WI). qPCR analysis was performed using the 7300 PCR Assay System and Step One Plus real-time PCR System (Applied Biosystems, Foster City, CA) with Taqman probes for 15-PGDH and GAPDH (HPGD, GAPDH; Applied Biosystems) and SYBR green PCR master mix (Applied Biosystems) for hPTEN using primers specific for hPTEN, sense 5′-CAGGACCAGAGGAAACCTCA-3′ and antisense 5′-GCTAGCCTCTGGATTTGACG-3′. For endogenous miRNA detection, 10 ng of total RNA was converted to cDNA using the Taqman microRNA reverse transcription kit (Applied Biosystems) with stem-loop miRNA primers specific for mature hsa-miR-21, and the small nuclear protein RNU6B (U6) control for normalization (Applied Biosystems). qPCR detection of miRNAs was performed using Taqman probes designed for miR-21 and U6 (Applied Biosystems) except in miR-21 sponge experiments. In these experiments, MiScript II Reverse Transcription Kit (Qiagen, Germantown, MD) was used for cDNA synthesis. miR-21 forward primer 5′-TCAGTAGCTTATCAGACTGATG-3′ was used in conjuction with a universal reverse primer (Qiagen).

### Protein, PGE_2_, and cell proliferation analysis

Cells were washed with PBS and lysed in RIPA buffer (50 mM Tris at pH 8.0, 150 mM NaCl, 1% Nonidet P-40, 0.5% sodium deoxycholate, 0.1% SDS, 0.1% protease inhibitor). 25 μg of protein were loaded onto 10% SDS-PAGE gels and transferred onto PVDF membrane (VWR, Radnor, PA). Western blots were performed using antibodies against 15-PGDH (HPGD; HPA005679; Sigma–Aldrich, St. Louis, MO), anti-Luciferase (Promega, Madison, WI), COX-2 (160112, Cayman Chemical, Ann Arbor, MI), and PTEN (9559, Cell Signaling Technology, Danvers, MA). Membranes were stripped and re-probed using β-actin (Clone C4; MP Biomedicals, Solon, OH), tubulin (HRP-66031, Proteintech, Rosemont, IL) or GAPDH (HRP-60004, Proteintech, Rosemont, IL). antibodies. Cells transfected with luciferase reporter constructs were lysed in reporter lysis buffer (Promega, Madison, WI) and assayed using the Luciferase Assay System (Promega). Reporter gene activities were normalized to total protein; all results represent the average of triplicate experiments. Prostaglandin E_2_ (PGE_2_) levels and prostaglandin (PG) levels in cell culture media were analyzed by PGE_2_ ELISA (R&D Systems, Minneapolis, MN), and Prostaglandin screening EIA kit (Cayman), respectively. Media was removed and cells were incubated for 20 min with serum-free media containing 10 μM arachidonic acid (Cayman) in serum-free media. Relative PGE_2_ and PG levels were normalized to total protein levels and are an average of three experiments. WST-1 Cell Proliferation Assay (Cayman Chemical, Ann Arbor, MI) was performed in 96-well plates per the manufacturer’s protocol, normalized to total protein, and represent the average of 3 biological replicates.

### miRNA Ribonucleoprotein immunoprecipitations

Immunoprecipitation of miRNA ribonucleoprotein complexes (mRNP-IP) was performed as described^71^, using a polyclonal anti-HA antibody (Santa Cruz Biotechnology, Santa Cruz, CA, USA) or control IgG pre-coated to protein A/G PLUS agarose (Santa Cruz Biotechnology). Total RNA was isolated from immunoprecipitates using 1 ml Trizol per IP reaction and Taqman or SYBR green qPCR analysis of mRNA in RNP-IP samples was performed as described above.

### Human tissue samples

Human colon tumors and histologically normal tissue were obtained from the Center for Colon Cancer Research (CCCR) Tissue Biorepository at the University of South Carolina (USC) with oversight and approval from the USC Institutional Review Board. Primary colorectal cancers were collected at the time of surgery by the CCCR Biorepository and immediately snap-frozen in liquid nitrogen and stored at −80°. Two independent pathologists confirmed diagnosis of all samples used in the study. Total RNA was isolated using Trizol from approximately 50 mg of tissue and converted to cDNA as described above. The quality of the resulting cDNA were determined for each sample by quantitative real-time PCR using GAPDH Taqman probes (for mRNA quality) and RNU6B (U6 RNA) primers (for small RNA quality). Samples were consented for use in biomedical research at the time of surgery. All the tissue samples and associated data obtained from the biorepository are fully deidentified.

### Bioinformatic & statistical analysis

The Cancer Genome Atlas (TCGA) was mined using the TCGA-assembler 2 R software package^72^. Colorectal (COAD) RNA-Seq (gene.normalized_RNAseq, gene_RNAseq)and miR-Seq (mir_GA.hg19mirbase20, mir_HiSeq.hg19.mirbase20) was downloaded by TCGA-assembler 2 and analyzed on R using internal lab written software. Inquiries about lab written code can be emailed to carollutzlab@gmail.com. The data are expressed as the mean +/− SEM. Student’s t-test and one-way ANOVA were used to determine significant differences. Where indicated, the Mann-Whitney U test was used to determine statistical significance. Inverse correlation studies used the Pearson product-moment correlation coefficient (PMCC) to determine the correlation value, r, and *P*-value was determined by using the correlation value, r, and the sample size. *P*-values less than 0.05 were considered significant.
